# Foreign Intelligence

**Published:** 1864-02

**Authors:** 


### FOREIGN INTELLIGENCE.

Treatment of Drowning.—The following rules have been just issued by the Royal Humane Society. They are stated to be "the results of the labors of the committee of the Royal Medical and Chirurgical Society of London."

Directions for Restoring the Apparently Dead.—I. *If from Drowning or other Suffocation or Narcotic Poisoning.*—Send immediately for medical assistance, blankets, and dry clothing; but proceed to treat the patient *instantly*, securing as much fresh air as possible.

The points to be aimed at are: first, and immediately, the restoration of breathing; and, secondly, after breathing is restored, the promotion of warmth and circulation.

The efforts to restore life must be persevered in until the arrival of medical assistance, or until the pulse and breathing have ceased for at least an hour.

Treatment to Restore Natural Breathing.—*Rule* 1. *To Maintain a Free Entrance of Air into the Windpipe.*—Cleanse the mouth and nostrils; open the mouth; draw forward the patient's tongue, and keep it forward; an elastic band over the tongue and under the chin will answer this purpose. Remove all tight clothing from about the neck and chest.

*Rule* 2. *To Adjust the Patient's Position.*—Place the patient on his back on a flat surface, inclined a little from the feet upwards; raise and support the head and shoulders on a small firm cushion or folded article of dress placed under the shoulder-blade.

*Rule* 3. *To Imitate the Movements of Breathing.*—Grasp the patient's arms just above the elbows, and draw the arms gently and steadily upwards, until they meet above the head (this is for the purpose of drawing air into the lungs); and keep the arms in that position for two seconds. Then turn down the patient's arms, and press them gently and firmly for two seconds against the sides of the chest (this is with the object of pressing air out of the lungs. Pressure on the breast bone will aid this.)

Repeat these measures alternately, deliberately, and perseveringly, fifteen times in a minute, until a spontaneous effort to respire is perceived, immediately upon which cease to imitate the movements of breathing, and proceed to induce circulation and warmth (as below).

Should a warm bath be procurable, the body may be placed in it up to the neck, continuing to imitate the movements of breathing. Raise the body in twenty seconds in a sitting position, and dash cold water against the chest and face, and pass ammonia under the nose. The patient should not be kept in the warm bath longer than five or six minutes.

*Rule* 4. *To Excite Inspiration.*—During the employment of the above method, excite the nostrils with snuff or smelling salts, or tickle the throat with a feather. Rub the chest and face briskly, and dash cold and hot water alternately on them.

The above directions are chiefly Dr. H. R. Silvester's method of restoring the apparently dead or drowned, and have been approved by the Royal Medical and Chirurgical Society.

TREATMENT AFTER NATURAL BREATHING HAS BEEN RESTORED. —*Rule* 5. *To Induce Circulation and Warmth.*—Wrap the patient in dry blankets, and commence rubbing the limbs upwards, firmly and energetically. The friction must be continued under the blankets or over the dry clothing.

Promote the warmth of the body by the application of hot flannels, bottles or bladders of hot water, heated bricks, etc., to the pit of the stomach, the arm-pits, between the thighs, and to the soles of the feet. Warm clothing may generally be obtained from bystanders.

On the restoration of life, when the power of swallowing has returned, a teaspoonful of warm water, small quantities of wine, warm brandy and water, or coffee, should be given. The patient should be kept in bed, and a disposition to sleep encouraged. During reaction large mustard plasters to the chest and below the shoulders will greatly relieve the distressed breathing.

II. *If from Intense Cold.*—Rub the body with snow, ice, or cold water. Restore warmth by slow degrees. In these accidents it is highly dangerous to apply heat too early.

III. *If from Intoxication.*—Lay the individual on a bed with his head raised. The patient should be induced to vomit. Stimulants should be avoided.

III. *If from Apoplexy or Sunstroke.*—Cold should be applied to the head, which should be kept well raised. Tight clothing should be removed from the neck and chest. Stimulants should be avoided.

*Appearances which Generally Indicate Death.*—There is no breathing or heart's action; the eyelids are generally half closed; the pupils dilated; the jaws clenched; the fingers semi-contracted; the tongue appearing between the teeth, and the mouth and nostrils are covered with a frothy mucus. Coldness and pallor of surface increases. —*Brit. Med. Journ.*, Sept. 12, 1863.—*Medical News.*

